# Detection of Respiratory Disease Based on Surface-Enhanced Raman Scattering and Multivariate Analysis of Human Serum

**DOI:** 10.3390/diagnostics15060660

**Published:** 2025-03-08

**Authors:** Yulia Khristoforova, Lyudmila Bratchenko, Vitalii Kupaev, Dmitry Senyushkin, Maria Skuratova, Shuang Wang, Petr Lebedev, Ivan Bratchenko

**Affiliations:** 1 Department of Laser and Biotechnical Systems, Samara National Research University, 34 Moskovskoe Shosse, 443086 Samara, Russia; shamina.la@ssau.ru (L.B.); bratchenko@ssau.ru (I.B.); 2 Family Medicine Department, North-Western State Medical University Named after I.I. Mechnikov, 41 Kirochnaya Street, 191015 Saint-Petersburg, Russia; v.i.kupaev@samsmu.ru; 3 Department of Outpatient Care, Samara State Medical University, 89 Chapaevskaya Str., 443079 Samara, Russia; dimasen389@gmail.com; 4 Samara City Clinical Hospital №1 Named after N. I. Pirogov, 80 Polevaya Str., 443096 Samara, Russia; skuratova_m@mail.ru; 5 Institute of Photonics and Photon-Technology, Northwest University, #1 Xuefu Avenue, Xi’an 710127, China; swang@nwu.edu.cn; 6 Postgraduate Department, Samara State Medical University, 89 Chapaevskaya Str., 443079 Samara, Russia; palebedev@yahoo.com

**Keywords:** surface-enhanced Raman scattering (SERS), liquid biopsy, projection on latent structures with discriminant analysis (PLS-DA), classification model, chronic obstructive pulmonary disease (COPD), respiratory diseases

## Abstract

**Background/Objectives**: Chronic obstructive pulmonary disease (COPD) is a significant public health concern, affecting millions of people worldwide. This study aims to use Surface-Enhanced Raman Scattering (SERS) technology to detect the presence of respiratory conditions, with a focus on COPD. **Methods**: The samples of human serum from 41 patients with respiratory diseases (11 patients with COPD, 20 with bronchial asthma (BA), and 10 with asthma–COPD overlap syndrome) and 103 patients with ischemic heart disease, complicated by chronic heart failure (CHF), were analyzed using SERS. A multivariate analysis of the SERS characteristics of human serum was performed using Partial Least Squares Discriminant Analysis (PLS-DA) to classify the following groups: (1) all respiratory disease patients versus the pathological referent group, which included CHF patients, and (2) patients with COPD versus those with BA. **Results**: We found that a combination of SERS characteristics at 638 and 1051 cm^−1^ could help to identify respiratory diseases. The PLS-DA model achieved a mean predictive accuracy of 0.92 for classifying respiratory diseases and the pathological referent group (0.85 sensitivity, 0.97 specificity). However, in the case of differentiating between COPD and BA, the mean predictive accuracy was only 0.61. **Conclusions**: Therefore, the metabolic and proteomic composition of human serum shows significant differences in respiratory disease patients compared to the pathological referent group, but the differences between patients with COPD and BA are less significant, suggesting a similarity in the serum and general pathogenetic mechanisms of these two conditions.

## 1. Introduction

Respiratory diseases affect the airways and other structures of the lungs. One of the most common types of respiratory diseases is chronic obstructive pulmonary disease (COPD) and bronchial asthma (BA). COPD is a heterogeneous lung syndrome characterized by chronic respiratory symptoms (shortness of breath, cough, sputum production, and/or exacerbations) due to abnormalities in the airways (bronchitis, bronchiolitis) and/or alveoli (emphysema) that cause persistent and often progressive air-flow obstruction [1]. According to the World Health Organization (WHO), COPD is the third leading cause of death worldwide, causing 3.23 million deaths in 2019 [1]. Usually, the main risk factors, such as smoking and ambient particulate matter pollution, gradually affect the human body and cause a slow development of the disease, without pronounced clinical symptoms in the early stages.

The difficulties in diagnosing COPD are due to the lack of widely accepted and well-established specific biomarkers for the disease. At present, the only “marker” widely accepted for diagnosing COPD is forced expiratory volume in one second (FEV_1_), which is measured by spirometry [2]. However, this marker has low sensitivity for detecting early pathological changes in the disease and can be misinterpreted [3,4]. For instance, the analysis of FEV_1_ can be problematic in older patients as the normal aging process can affect lung volumes. Early identification of the disease can dramatically improve the global health of the population, as timely treatment and therapy can change the outcome. To overcome problems in COPD diagnosis, many studies are focused on developing new, precise approaches [5,6] and reliable indicators [7] that can estimate and recognize biochemical changes in patients’ samples in a pathological COPD state.

In recent years, optical diagnostics based on a combination of optical methods and machine learning techniques have become increasingly popular as a support for diagnostic approaches [8,9,10,11,12]. Among various optical methods, Raman spectroscopy has emerged as a leading method due to its high sensitivity and ability to solve a wide range of diagnostic problems [10], from identifying cancer cases [13,14] to detecting neurodegenerative diseases [15,16,17]. The Raman spectrum of a biological sample, such as tissue, biofluid, or cell strain, contains a set of peaks that are caused by the chemical bonds of the molecular components present in the sample [18]. In liquid optical diagnostics, attention has been focused on the application of Surface-Enhanced Raman Scattering (SERS), which offers excellent molecular specificity, ultra-sensitivity, and the ability to detect specific analytes at low concentrations. SERS is a powerful technique based on Raman spectroscopy that uses plasmonic nanostructures to amplify the Raman signal by a factor of thousands [11,19,20,21,22]. This technique, combined with advanced statistical analysis and machine or deep learning methods, allows us to obtain more accurate and detailed information about the biochemical composition and properties of biomolecules in complex biological environments.

Recently, a number of research groups have reported promising results from the application of Raman-based techniques, particularly SERS, in the diagnosis of respiratory diseases by analyzing saliva, blood, and serum human samples. Ullah et al. [23] successfully demonstrated Raman spectroscopy combined with chemometric techniques for screening asthma disease by analyzing human blood serum. Widely developing diagnostic modalities for SARS-CoV-2 infection based on Raman spectral data analysis with machine learning and deep learning algorithms have also been presented [24,25,26,27]. Additionally, preliminary attempts have demonstrated the potential success of the SERS approach for COPD monitoring through analysis of human saliva samples by Carlomango et al. [5].

Nevertheless, COPD diagnostics using SERS are not well studied. The occurrence of COPD is mainly caused by chemical oxidative stress. Due to oxidative metabolism disorders, cells in the lungs (macrophages and neutrophils, etc.) actively participate in biochemical reactions [28]. It is known that imbalances in lipid metabolism [29] and other biochemical processes are correlated with the severity of COPD. Therefore, the application of SERS can identify the distinctive features of COPD during the analysis of blood biochemical changes reflected in the registered spectral patterns. Although blood sampling is an invasive method, blood is the universal environment of the human body and reflects pathological processes in all organs. Therefore, blood serum is an ideal candidate for spectral analysis to detect COPD.

In clinical practice, the diagnosis of COPD is complicated by overlapping signs and symptoms with chronic heart failure (CHF). In both, dyspnea is the main clinical symptom. Moreover, the correct diagnosis can be difficult because respiratory and heart conditions often occur together in older patients [30,31]. Thus, it is important to study the possibility of detecting COPD among a comparable pathological referent group.

This paper aims to conduct an in vitro analysis of blood serum spectral features using SERS and advanced statistical analysis in order to detect cases of COPD. We will use Partial Least Squares Discriminant Analysis (PLS-DA) to classify COPD and BA using SERS spectra, as well as to predict the type of respiratory state and to identify unknown tested cases. Additionally, we will analyze serum samples from patients with CHF in order to differentiate them from those with COPD.

## 2. Materials and Methods

### 2.1. Experimental Setup and Spectra Measurement

Achieving surface enhancement of the Raman signal of serum was realized using a structured silver surface. As a basis for silver structures, a silver colloid was prepared by reducing silver nitrate (AgNO_3_) from an aqueous solution with sodium citrate (Na_3_C_6_H_5_O_7_) at a temperature of 95 °C for 20 min. The prepared silver colloidal solution is characterized by spherical nanoparticles with a diameter of 30–40 nm. In order to form more complex and larger structures, the resulting colloidal solution with a volume of 20 mL was poured onto aluminum foil with an area of 75 mm × 25 mm and dried at room temperature until completely dry. The structured surface obtained in this way consists of agglomerates of spherical particles with a size of about 200 nm. The method used to prepare silver surfaces and their characteristics is described in more detail in the previous work [32]. It should be noted that the Raman spectrum of the used silver structured surface on the foil is characterized by the absence of clearly defined Raman peaks. Therefore, there is no need to take into account the spectral contribution of the plate to the SERS characteristics of the serum in subsequent multivariate analysis. To record spectral characteristics, each serum sample in a volume of 2 μL was applied to a structured silver surface and dried at room temperature for 30 min. The SERS serum spectra were collected using an experimental setup consisting of a spectrometric system (EnSpectr R785, Spektr-M, Chernogolovka, Russia) and a microscope (ADF U300, ADF, Hangzhou, China) with 50× LMPlan Objective. The SERS spectra were registered in the 517–1913 cm^−1^ spectral range under 785 nm excitation radiation. The diameter of the laser spot at the focus was 5 µm. The serum samples were analyzed at the laser power of 10 mW. The spectra were recorded with the exposure time of 4 s 4 times. The background component of the stand optical system was automatically subtracted from the subsequent recorded serum spectra using the algorithm built in the EnSpectr software (123123123 app. version).

### 2.2. Blood Serum Samples

In this study, we analyzed the serum of patients with respiratory diseases and CHF from the Samara City Clinical Hospital No. 1 named after N. I. Pirogov and the Samara Regional Clinical Hospital named after V. D. Seredavin. We only included patients from similar age groups in this study. Therefore, we analyzed blood serum samples from 144 participants, including 41 patients with respiratory conditions and 103 with CHF. The number of patients and registered spectra are provided in Table 1. The respiratory group included 11 cases of COPD, 20 cases of BA, and 10 cases of COPD&BA. Patients with bronchial obstruction were diagnosed using the GOLD and GINA criteria. Exclusion criteria were taking hormonal medications. This study included patients with BA who had late-onset diseases (after 30 years) and were predominantly non-atopic with an unsatisfactory level of control. Spirometry results were assessed after a bronchodilator test using a Vitalograph 2120 spirometer. Patients with COPD&BA had a long history of smoking against the background of atopy, with a positive bronchodilator test and an FEV_1_/FVC < 0.7. All patients with COPD corresponded to the 2nd and 3rd degree of disease severity (moderate and severe) by FEV_1_ with a mixed phenotype (bronchial and emphysematous). According to the GOLD 2023 classification, all patients corresponded to group E, i.e., had more than 1 exacerbation per year or had 1 exacerbation that resulted in hospitalization. Table 2 provides information on patients with BA, COPD, and COPD&BA.

CHF was caused by chronic ischemic heart disease in all 103 of the included patients in this group. Concomitant arterial hypertension occurred in 96% of them and previous myocardial infarction occurred in 64%. According to the AHA/ACC/HFSA 2022 Guidelines, all those included in the CHF group had overt heart insufficiency of “C” stage, whilst II NYHA class was seen in 89 patients (86%) and III class was seen in 14 patients (13%). Left ventricular ejection fraction (LVEF) <40% was seen in 56%; LVEF 41–49% was seen in 34%; and LVEF >50% was seen in 10% of patients.

The serum samples were collected from the patient in the morning, in the fasting state, and were placed in sterile test-tubes followed by freezing at the temperature of −16 °C. The study protocols were approved by the ethical committee of Samara State Medical University (protocol no. 268, 11 September 2023). All involved patients agreed to participate in this study by written informed consent.

### 2.3. Spectra Preprocessing and Multivariate Statistical Analysis

The spectra in the 517–1913 cm^−1^ region were preprocessed by the smoothing technique using the Savitzky–Golay filter (15 filter window width, 1 order of polynomial used for smoothing, and 0 orders of derivative to take into account), removal of background was achieved using the polynomial method (15th-degree polynomial), and normalization was achieved using the standard normal variate method (SNV). Each preprocessed SERS serum spectrum was a discrete set of 1950 spectral intensity values at 517–1913 cm^−1^ wavenumbers (predictors). Prior information on the belonging of each sample to a specific disease group was known. This allowed us to build statistical models to classify samples and to estimate their performance. Multivariate statistical analysis was performed on human SERS serum spectra to build binary classification tasks relevant to COPD detection:

(1) “respiratory diseases (COPD + BA+ COPD&BA) vs. pathological referent group (CHF)”;
(2) “COPD vs. BA”.

The classification algorithm was based on the projection on latent structures with discriminant analysis (PLS-DA). PLS reduces the spectral dimensionality to a smaller number of new orthogonal components called latent variables (LVs). LVs are linear combinations of the original spectra and are defined based on the variability of the spectral values. This variability is correlated with the different classes of spectra, meaning that only those spectral features that distinguish between the classes under study are used.

**Figure 1:**
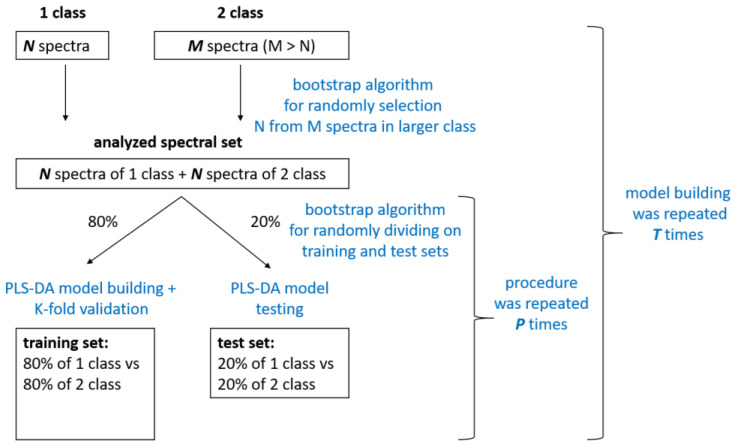
Scheme of PLS-DA model building procedure.

Figure 1 shows the scheme of the PLS-DA model building process. In accordance with Table 1, the number of SERS spectra in each class differed in the classification tasks considered. We used the bootstrap algorithm [33] to randomly select a subset of SERS spectra from the larger class in order to obtain classes with comparable sizes. Then, 80% of these subsets were selected again by the bootstrap algorithm and used as the training set for building the PLS-DA model. The model’s performance was validated using K-fold cross-validation. The CV procedure helped to determine the optimal number of LVs when building the model, to avoid overfitting. We selected the optimal number of LVs based on the first local minimum in the root mean square error (RMSE) plot, which was calculated for different numbers of LV and CV predictions. The model performance was evaluated on the test set, which was the remaining 20% of the selected spectral subset. It should be noted that the division of the data into training and test subsets was achieved in such a way that spectra from the same patient either fell into the training or the test set. This allowed us to assess the performance of the model on new, unseen data that had not been analyzed by the model during training. The process of dividing the spectral data into training and testing subsets was repeated *P* times. Then, PLS-DA model training was repeated *T* times. It should be noted that at each iteration of model training, SERS spectra selected by the bootstrap algorithm were treated as a new observation. This algorithm for performing multiple modeling iterations allowed us to evaluate the reliability and consistency of the results obtained from PLS-DA modeling on a limited experimental dataset.

For each PLS-DA model, we calculated and analyzed the variable importance in projection (VIP) scores [34] to explain the informative SERS peaks in the model. The results of the serum SERS spectra differentiation were visualized using the receiver operating characteristic (ROC) curves. For quantitative analysis, the area under the curve (AUC) was calculated. Statistical analysis was carried out using the MDAtools package available within the R studio software (v4.3.2) [35].

## 3. Results and Discussion

### 3.1. SERS Serum Spectra

**Figure 2:**
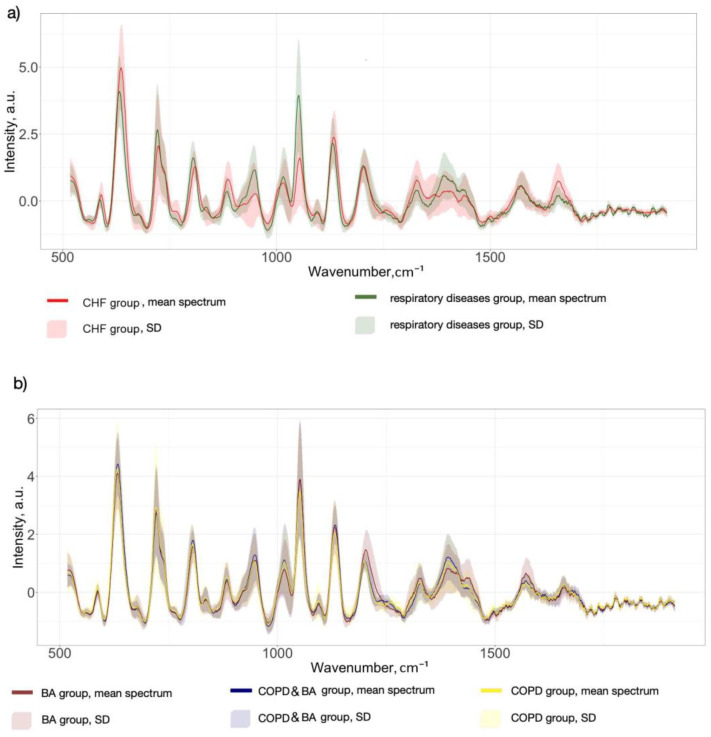
The mean spectra with standard deviation (SD) of human serum of patients with different pathologies: (**a**) respiratory diseases vs. CHF; (**b**) different types of respiratory diseases: COPD, BA, and COPD&BA.

SERS spectra were collected from serum samples from two different groups of patients: 143 spectra came from the respiratory diseases group and 309 spectra came from the CHF group. Figure 2 shows the mean SERS spectra of serum samples for each pathology group and for each respiratory disease separately. Mean spectra were formed by averaging all the spectra of each disease. As we can see, the SERS serum spectra of all disease groups consist of a set of prominent Raman peaks in 500–1800 cm^−1^ wavenumbers assigned to the different serum constituents, with the following possible associations [4,36]: 587 cm^−1^ (Phosphatidylinositol in lipids), 638 cm^−1^ (uric acid), 724 cm^−1^ (δ(C–H) of adenine, coenzyme A, DNA/RNA), 805 cm^−1^ (ν(C–C–O) of L-Serine, glutathione), 890 cm^−1^ (δ(C–O–H) or ring bending of Tryptophan, glutathione, tryptophan, D-(C)-galactosamine), 947 cm^−1^ (C-C stretching of proteins), 1008 cm^−1^ (Phenylalanine), 1051 cm^−1^ (Glycogen in Carbohydrates), 1132 cm^−1^ (ν(C–N) in D-Mannose), 1207 cm^−1^ (ring vibrations in L-Tryptophan, phenylalanine), 1329 cm^−1^ (Torsion mode CH_2_ in lipids), 1390 cm^−1^ (C−N, C−H group, ω(CH_3_), CH_2_ wagging in lipids), 1442 cm^−1^ (CH_2_/CH_3_ deformations in proteins and lipids), 1568 cm^−1^ (DNA/RNA bases), and 1657 cm^−1^ (ν(C-O), Amide I).

The highlighted spectral differences were observed between the mean spectra of the respiratory disease group and CHF. In particular, for the respiratory diseases group, the SERS signal intensities at 724, 947, 1051, and 1390 cm^−1^ were higher, while the peaks at 638 and 1657 cm^−1^ showed a decrease compared to the opposite group (Figure 2a). It is interesting to note that the serum SERS spectra of patients with different types of respiratory diseases, as presented in Figure 2b, were more similar in terms of spectral intensity values. Spectral differences between COPD and BA cases were observed at several bands, with higher intensities at the 638, 1008, and 1392 cm^−1^ bands for COPD mean spectra and at the 1051, 1207, and 1568 cm^−1^ bands for BA mean spectra. However, it is difficult to visually identify specific patterns in the spectral signal for COPD cases. Due to the complex biochemical composition of serum, the standard deviation of Raman intensities overlaps at the same peaks between the values of different patient groups. Additionally, it is important to note that the serum composition does not fundamentally change in different diseases; the ratios of different functional groups and backbone chains, for example, in proteins, lipids, and nucleic acids are changed. To identify significant spectral differences between groups of patients and use them for differentiation, multivariate statistical methods should be applied. In this study, the PLS-DA approach was applied to build two classification models: respiratory diseases vs. CHF and COPD vs. BA.

### 3.2. PLS-DA Classification Models

PLS-DA models were built on the training set for each classification task and tested on the test set. The performance of the classification models based on PLS-DA is presented in Table 3, where the sensitivities, specificities, and accuracies are shown in terms of mean and minimum and maximum values achieved across all modeling folds.

The first model (I) was a binary classification to differentiate between all respiratory diseases and CHF cases (pathological referent group). We want to emphasize the importance of forming the pathological referent group correctly. It consists of patients with a CHF of the same age. As we can see from Table 1, the age ranges and mean ages are almost the same for the group with respiratory diseases and CHF patients, which eliminates the influence of age on the classification results.

**Figure 3:**
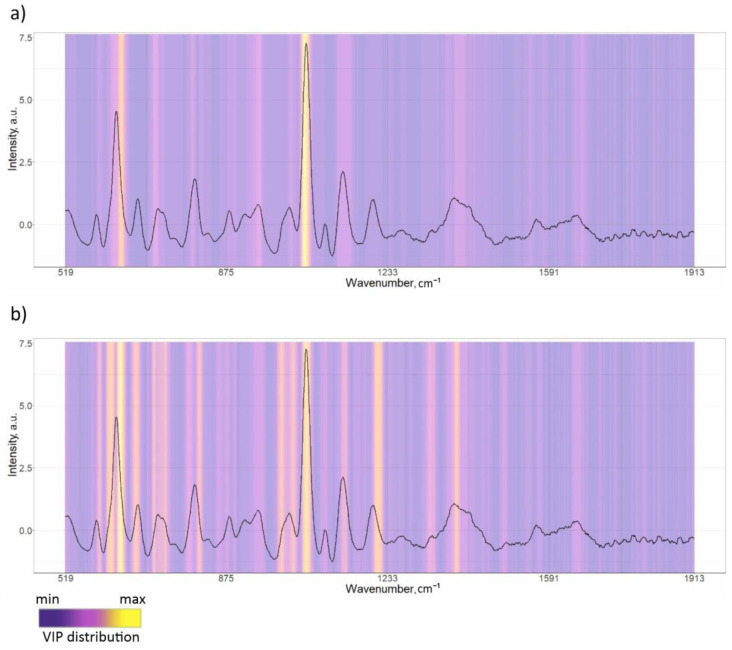
VIPs’ distribution for constructed PLS-DA models: (**a**) “respiratory diseases vs. CHF”; (**b**) COPD vs. BA.

The spectra of 41 patients from the respiratory pathology group and 41 patients from the CHF were randomly selected from the total spectral set using the bootstrap algorithm. This allowed us to build a model on balanced groups. The analyzed spectral set was divided into training and testing sets multiple times (*P* = 5). At each fold, training was performed on the training set, which consisted of 196 spectra (80% of the spectra selected from 82 patients). Model building iterations were repeated *T* = 5 times; thus, we averaged and analyzed the model results for 25 different folds. A total of 5 to 7 LVs were used to train the model in each of the 25 folds, according to the RMSE criterion. Figure 3a shows the mean VIPs for the 25 folds, with a gradient fill overlayed on the serum SERS spectrum, where purple corresponds to the minimum useful contribution of a spectral band in the model and yellow corresponds to the maximum contribution (scale shown in Figure 3). In accordance with the VIP distribution, the 630–650 cm^−1^ band with a maximum at 638 cm^−1^ (uric acid) and the 1065–1081 cm^−1^ band with a maximum at 1051 cm^−1^ are characterized by significant contributions in building the PLS-DA model. For this study, the mean values of sensitivity, specificity, accuracy, and ROC AUC over five models were observed as 0.94, 0.95, 0.95, and 0.97, respectively, during training (Table 3).

At each fold, after the classification was performed on the training set, its capability was tested on the test set, which consisted of 50 spectra (20% of the total number of spectra). We calculated the average sensitivity, specificity, and accuracy over 25 iterations for each test set of all folds. As we can see in Table 3, the sensitivity and specificity values of the test set have slightly higher variances compared to the training set. However, the minimal and mean accuracy values of 0.82 and 0.92 over 25 iterations prove the high performance of differentiating between respiratory diseases and CHF. These results show that the biochemical changes during pathology are reflected in the SERS spectral profile of serum and depend on the type of disease, allowing us to identify respiratory diseases among pathological referent patients.

After identifying respiratory diseases, a second classification model was developed to classify the types of respiratory diseases, specifically to differentiate between COPD and BA cases. A total of 33 spectra from 11 patients with COPD and 33 spectra from 11 patients with BA were randomly selected using the bootstrap algorithm to train and test the model. The model was trained using 52 spectra and tested using 14 spectra selected randomly by the bootstrap algorithm. This process was repeated *P* = 3 times to divide the analyzed spectra into training and test sets. The model building procedure was repeated *T* = 3 times, allowing us to achieve and average the results for nine different folds. In accordance with the mean VIP distribution averaged over nine folds in Figure 3a, the most dominant changes were observed at 638, 724, 947, 1025, 1051, 1329, and 1395 cm^−1^ peaks, amongst others.

The results of the PLS-DA statistical model for differentiating COPD from BA cases based on serum SERS spectra are presented in Table 3. We observed a significant decrease in sensitivity, specificity, and accuracy values for the test set compared to the training set. In addition, the mean ROC AUC was 0.93 for the training set and 0.72 for the test set, indicating that the model did not perform as well when verifying new cases.

In total, the “COPD vs. BA” (II) model shows much lower performance compared to the “respiratory diseases vs. CHF” (I) model, with mean accuracy values of 0.95 for the training set and 0.92 for the test set for model I, and 0.89 and 0.61, respectively, for model II. We can confidently claim that the achieved performance is not accidental, as the mean test ROC AUC of 25-folds equals 0.97 with a relatively small variance of 0.0029, indicating the stability of the classification model. Therefore, it can be argued that there are biochemical changes in human serum that can be “caught” by SERS, allowing for the identification of respiratory diseases from other conditions. On the other hand, the performance of the “COPD vs. BA” model was tested on a test set in order to check the reliability of its diagnostic capabilities. However, the results were not satisfactory. The ROC AUC values showed high variability, ranging from 0.53 to 1.0, for nine different folds of the test set. Furthermore, differences in the spectral bands, highlighted by VIP as being important (yellow gradient fill in Figure 3b), demonstrate the absence of specific bands and changes in characteristics of COPD compared to BA.

VIP analysis of the “respiratory diseases vs. CHF” model prominently highlights specific spectral changes between the two groups in Raman bands at 638 and 1051 cm^−1^, which were also observed in the differences in mean spectra. Assigning spectral bands in serum spectra to specific compounds is a complex task due to the multi-component nature of serum. While a particular Raman band can be associated with a specific compound, its contribution is not exclusive. For instance, the Raman band at 638 cm^−1^ has been linked to uric acid based on comparisons with known metabolite SERS signals, as detailed in the literature [37,38,39]. Increased uric acid levels are linked to various biological processes, such as oxidative stress and inflammation. Several epidemiological studies [40,41,42] suggest a possible association between elevated uric acid levels and certain diseases such as kidney diseases and cardiovascular disease, although the exact nature of this relationship is still uncertain. In our study, we also found that SERS signals from uric acid at 638 cm^−1^ in the serum of CHF patients showed a higher intensity compared to serum samples of patients with respiratory diseases. The relationship between COPD and uric acid levels has been discussed in relation to the effect of hyperuricemia on the progression of the condition. A paper by Sarangi [43] discussed the relationship between serum uric acid levels and the severity of COPD in patients. It is interesting to note that the study found that serum uric acid levels increased with increasing COPD severity. H. Yang et al. [44] have also reported that elevated serum uric acid levels were linked to reduced lung function. However, no significant impact of serum uric acid on lung function was observed in those without COPD. In the current study, we do not have sufficient data on the uric acid levels in serum samples to verify this assumption. Nevertheless, the comparison of the 638 cm^−1^ peak intensities between our samples with the second and third degree of COPD severities was not statistically significant (*p* > 0.05). Peaks related to glycogen at 1051 cm^−1^ are more prominent in the respiratory diseases’ mean spectrum compared to the CHF group, possibly indicating metabolic alterations occurring in COPD. Acute hyperglycemia is associated with poor outcomes in patients with various diseases and data on hyperglycemia and COPD in particular are known [45].

Difficulties in differentiating between BA and COPD are also seen in clinical practice. This could be due to the lack of specific biomarkers that could identify these conditions, despite their different pathogenesis. Several studies [6,46] have studied the role of biomarkers such as periostin, IL-18, CCL-18, and others in the systemic inflammation of these disorders, but there have not been any significant differences reported between BA and COPD. It is possible that neutrophilic inflammation plays a role in the pathogenesis of both conditions, which results in the complexity of differential diagnosis [47]. However, the relationship between neutrophil numbers in blood serum and registered spectral data have not been analyzed in this study.

To our knowledge, only a few papers have discussed the study of COPD cases using Raman-based techniques [4]. This is a very interesting area of research due to its clinical relevance and many aspects require detailed analysis. The main goal of developing an optical diagnostic method is to identify specific changes and spectral markers during the progression of COPD in order to explain the results of spectral analysis. C. Carlomango et al. [5], for example, performed a comparative Raman analysis of pathological saliva samples from COPD patients and healthy individuals. They found informative spectral differences between the two groups with changes in lipid and carbohydrate regions in the 500–600 and 1250–1350 cm^−1^ ranges, as well as changes in proteins, carbohydrates, and nucleotide vibrational modes in the 900–950 and 1100–1200 cm^−1^ regions. In this study, we found that significant spectral changes associated with the presence of respiratory diseases could be related to the 638 cm^−1^ band of uric acid and 1051 cm^−1^ of glycogen. However, the changes associated with COPD cases, in contrast to those of BA, at 638, 724, 947, 1025, 1051, and 1329 cm^−1^ bands, have so far prevented us from proposing a model with high accuracy when verifying this.

The main limitation of this study is the small number of patients with respiratory diseases, particularly those with COPD. To overcome this problem, we created balanced groups of patients with respiratory disease and CHF using the bootstrap algorithm (see Figure 1) to build classification models. This approach allowed us to ensure an equal number of spectra in each group, which prevented imbalances in the data and led to the creation of reliable statistical models. Nevertheless, to verify the results and conclusions of this preliminary study, it is necessary to perform additional tests on a larger sample size of patients. Moreover, the heterogeneity of BA and COPD encompasses multiple phenotypes and endotypes, which are crucial for diagnosis and treatment. Therefore, substantial work on expanding the sample size considering the various phenotypes of respiratory diseases is required before a diagnostic method based on SERS can become viable.

The other limitation is that the differences in SERS spectra reflect nonspecific changes in human serum associated with the analyzed diseases, especially COPD. The molecular components associated with the informative spectral peaks represent broad functional groups of chemicals and the estimation of these changes may be overly general. Additionally, without the results of laboratory biochemical analysis of the serum, it is difficult to draw definitive conclusions about the possible causes of the observed differences. Therefore, a further area of study for the future is to combine SERS with precise biochemical analysis in clinical laboratories on the same samples to compare results of spectral analysis with quantitative chemical profiles of human serum.

## 4. Conclusions

This study demonstrates the identification and differentiation of respiratory diseases using SERS analysis of human serum samples. PLS-DA was used to build two binary classification models: “respiratory disease group vs. control group” and “COPD vs. BA” based on informative spectral differences. A reliable PLS-DA model was achieved to differentiate all respiratory diseases, including COPD, BA, and COPD&BA, from the opposite group, which includes patients with CHF patients, with a mean accuracy of 0.95 during training. To validate the model’s performance, analysis of a test set was conducted, achieving a mean accuracy of 0.92. The high performance of this model reflects the different chemical properties of serum in patients with overt chronic respiratory and CHF. In the future, it is possible that the identified spectral differences may predict the progression of CHF in patients with COPD. A second statistical model was built to differentiate COPD cases from BA with an accuracy of 0.89 during training and a 0.61 accuracy at verification. The differences between COPD and BA patients are less significant, suggesting similarities in serum analytes, spectral similarities, and common pathogenetic mechanisms between these two conditions. Further studies in this area are needed. At the next stage, the approach could be optimized through the correlation between serum spectral characteristics and the main components associated with COPD, which may improve the description of COPD-related spectral features in the serum. In addition, it is necessary to evaluate the performance of the models on a larger experimental dataset in order to demonstrate the general applicability of the proposed approach as a screening method.

## Figures and Tables

**Table 1:** Summary of analyzed patient groups.

Group of Subjects	Number of Patients	Mean Age (Min–Max)	Total Number of Spectra
Respiratory diseases (COPD + BA + COPD&BA)	41 (21 male, 20 female)	61 (39–74)	143
Chronic heart failure (CHF)	103 (76 male, 27 female)	65 (43–74)	309

**Table 2:** Summary of patients with respiratory diseases.

	BA, *n* = 20	COPD, *n* = 11	COPD&BA, *n* = 10	*p*-Value		
	Mean ± SD			$p_{\mathrm{BA}-\mathrm{COPD}}$	$p_{\mathrm{BA}-\mathrm{ACOS}}$	$p_{\mathrm{COPD}-\mathrm{ACOS}}$
Smoker’s index (packs/years)	0.032 ± 1.66	14.46 ± 16.63	27.38 ± 12.33	0.001	0.001	0.012
Body mass index	28.37 ± 4.96	26.77 ± 3.72	29.20 ± 5.70	0.146	0.409	0.057
Experience of the BA, year	13.73 ± 8.77	–	9.15 ± 8.81	–	0.070	–
Experience of the COPD, year	–	5.50 ± 5.05	7.31 ± 4.75	–	–	0.289
IGS, μg/day	301.75 ± 258.98	102.50 ± 216.63	326.15 ± 261.71	0.002	0.729	0.008
The number of exacerbations per year	1.55 ± 0.75	1.69 ± 1.08	2.31 ± 2.06	0.728	0.273	0.525
ACT, scores	16.82 ± 5.71	–	13.15 ± 4.58	–	0.047	–
CAT, scores	–	20.47 ± 8.06	22.75 ± 5.40	–	–	0.494
FEV_1_ (%)	77.40 ± 20.45	53.55 ± 28.06	53.48 ± 15.24	0.006	0.002	0.956
FVC (%)	79.17 ± 20.69	74.66 ± 34.76	65.53 ± 15.26	0.632	0.051	0.505
FEV_1_/FVC	0.79 ± 0.09	0.62 ± 0.15	0.64 ± 0.13	0.001	0.005	0.720

IGS—inhaled glucocorticosteroids; ACT—Asthma Control Test; CAT—COPD Assessment Test; FEV_1_—forced expiratory volume in one second; FVC—forced vital capacity.

**Table 3:** Classification results.

Classification Models		Specificity Mean (Min–Max)	Sensitivity Mean (Min–Max)	Accuracy Mean (Min–Max)	ROC AUC Mean (Min–Max)
Respiratory diseases (COPD + BA+ COPD&BA) vs. CHF (pathological referent group)	Training set	0.95 (0.92–1.0)	0.94 (0.91–0.99)	0.95 (0.94–0.98)	0.97 (0.96–1.0)
	Test set	0.97 (0.86–1.0)	0.85 (0.70–1.0)	0.92 (0.82–1)	0.96 (0.85–1.0)
COPD vs. BA	Training set	0.92 (0.86–1.0)	0.86 (0.75–0.92)	0.89 (0.85–0.96)	0.93 (0.78–0.99)
	Test set	0.57 (0.17–1.0)	0.64 (0.0–1.0)	0.61 (0.1–1.0)	0.72 (0.53–1.0)

## Data Availability

The data presented in this study are available upon request from the corresponding author.
